# Anxiety, Loneliness, Drug Craving, and Depression Among Substance Abusers in Sichuan Province, China

**DOI:** 10.3389/fphar.2021.623360

**Published:** 2021-07-07

**Authors:** Xin Chen, Nan Qiu, Liang Zhai, Gui Ren

**Affiliations:** ^1^ Department of Sport and Health Sciences, Technical University of Munich, Munich, Germany; ^2^ Southwest University, Chongqing, China; ^3^ General and Experimental Psychology, Department of Psychology, Ludwig-Maximilians-Universität München, München, Germany; ^4^ College of Physical Education, Sichuan Agricultural University, Yaan, China; ^5^ College of Physical Education, Guizhou University, Guiyang, China

**Keywords:** anxiety, loneliness, drug craving, depression, substance abusers

## Abstract

Studies have reported that anxiety had a positive effect on depression among substance abusers in males. However, little is known about the mechanism underlying this relationship in substance abusers in males. The purpose of this study was to investigate the mediating effect of loneliness and drug craving between anxiety and depression in substance abusers in males. State-Trait Anxiety Inventory, The UCLA Loneliness Scale, Drug Craving Scale, and The Center for Epidemiologic Studies Depression Scale were employed into this study to investigate 585 substance abusers in males (age range of 20–58 years: M = 33.21, SD = 6.97). Structural equation modeling and the bootstrap approach were used to analyze the mediating effect of loneliness and drug craving on the relationship between anxiety and depression. The results indicated that: Loneliness had a significant positive correlation with anxiety (*r* = 0.37, *p* < 0.001) and depression (*r* = 0.49, *p* < 0.001); Drug craving had a significant positive correlation with anxiety (*r* = 0.28, *p* < 0.001) and depression (*r* = 0.39, *p* < 0.001). Loneliness and drug craving mediated the link between anxiety and depression. There was a significant positive correlation between substance abusers in male’s anxiety, loneliness, drug craving, and depression. Loneliness and drug craving had a significant mediating effect on the relationship between anxiety and depression. Therefore, it was suggested that substance abusers in male’s anxiety and depression might be improved and driven by decreasing loneliness and drug craving. Targeted interventions to decrease substance abusers in male’s loneliness and drug craving should be developed, implemented, and help them to recover.

## Introduction

WHO surveys show that depression is an increasingly important factor affecting physical and mental health in the 21st century (Holden, 2000). Approximately 300 million people worldwide suffer from depression, and about 800,000 people commit suicide owing to depression each year (WHO, 2018). By 2020, depression will replace cancer as the world’s second most common disease (Brundtland, 2001). According to the Global Burden of Disease survey, depression’s disease burden ranks first in terms of years of healthy life lost due to a disability, with a weight of about 10.3% (Smith, 2014); and it is extremely painful (Ledford, 2014; Herrman et al., 2019). Depression is a common and easily neglected mental illness. It has become a major factor of “mental disability” worldwide, especially among young and middle-aged individuals (Vos et al., 2017). Depression is also called a depressive disorder, a type of mood disorder characterized by significant and lasting depression. It has the characteristics of chronic, recurrent, persistent, and high suicide rates (American Psychiatric Association, 2013). Early detection of depression tendencies and mild depression could allow for timely and positive intervention, which could prevent further depression of depression, it is of great significance to find a rapid, objective, and accurate diagnosis of depression and the best treatment for affected individuals. Recently, many empirical studies have found that anxiety, loneliness, drug craving, and depression are significantly positively correlated and affect each other among different groups of people (Chen et al., 2017; Xiao et al., 2018; Wolitzky-Taylor and Schiffman, 2019; Chen et al., 2021; Dziedzic et al., 2021; Kara et al., 2021). This study intended to further explore the mechanism of loneliness and drug craving in the relationship between anxiety and depression in drug users. This was not only helpful for a deeper understanding of the mechanism of anxiety affecting substance abusers in male’s depression, but also for guiding the prevention and treatment of depression, helping avert the occurrence of psychological and behavioral problems, and aid in improving the drug users’ health.

The comorbidity of depression and anxiety has become a current hotspot in global psychiatry circles and is gradually attracting the attention of scholars in different fields (Wang Y. H. et al., 2020; Katz et al., 2020; Santini et al., 2020). Anxiety is a common mental health problem, and individuals in anxiety often feel irritable and worried (Bekker et al., 2003). Some have found that social anxiety and depression accompany and affect each other (Hamilton et al., 2016; Klemanski et al., 2017). Anxiety is a factor that can more likely increase the risk of depressive symptoms and is an important predictor of depressive symptoms (Beesdo et al., 2007; Ottenbreit et al., 2014). A multiple pathways model to anxiety-depression comorbidity proposed by Cummings et al. (2014), showed that anxiety occurred in both depressive symptoms and depression. In addition, some experimental studies found that teenagers with anxiety disorders were three times more likely to show depressive symptoms than teenagers without them (Kessler et al., 1999). The relationship between anxiety and depression among different groups has been confirmed. Anxiety can positively predict depression, and simultaneously, there is a significant positive correlation between depression and anxiety (Beiter et al., 2015; DeVito et al., 2019; Yang et al., 2019). Some longitudinal studies found that as anxiety level increased, the individuals’ depression level also increased, which confirmed the clinical comorbidity of anxiety and depression (Wang Y. et al., 2020). Based on the above discussion, this study explored the relationship between anxiety and depression in substance abusers in males and its internal mechanism. Furthermore, it hypothesized that there was a significant positive correlation between anxiety and depression (H1).

Loneliness refers to a negative psychological experience that occurs when the interpersonal communication and intimacy desired by an individual cannot be satisfied; it is often accompanied by multiple negative emotions (Weiss, 1987; Asher and Paquette, 2003). Loneliness, anxiety, and depression are common health problems for individuals (Moeller and Seehuus, 2019; Igbokwe et al., 2020). If the individual has a high level of loneliness, it will cause depression and anxiety (Cacioppo et al., 2015). Some scholars have suggested that loneliness and depression have similar characteristics, but their performance was different (Heinrich and Gullone, 2006; Erzen and Çikrikci, 2018). Loneliness is a common life experience and changes with age (Nicolaisen and Thorsen, 2017). Some studies demonstrated through empirical research that loneliness, depression, and anxiety are inextricably linked (Richardson et al., 2017; Michalska da Rocha et al., 2018). A significant correlation analysis found a positive correlation between loneliness, anxiety, and depression (Jing et al., 2012; Ostovar et al., 2016; McHugh Power et al., 2020). A previous study showed loneliness had a significant mediating effect on anxiety and depression (Ebesutani et al., 2015). Based on the existing literature, it appeared there was a close relationship between loneliness and anxiety and depression, which may be a mediator variable worth considering. Therefore, this study hypothesized that loneliness mediates the relationship between anxiety and depression in substance abusers in males (H2).

Drug addiction is a physiological, psychological, biological, and social disorder. It presented huge challenges to social governance and public safety and harms the health of drug users and their families (Cami and Farré, 2003; Cardinal and Everitt, 2004; Bachi et al., 2017). Individual drug addiction is affected by many factors, among which drug craving is one of the most important factors leading to drug addiction (Self, 1998). Drug craving is a strong and uncontrollable desire for drug users to feel the effects of psychoactive substances they have experienced and the driving force to continue using drugs regardless of serious consequences (Bo et al., 2007). The increase in drug craving will reduce the pleasure that users get from drugs (Robinson and Berridge, 1993), thereby increasing the experience of anxiety and depression. Drug users with low drug cravings have relatively low levels of anxiety and depression (De Los Cobos et al., 2011). The self-discrepancy theory believes that when an individual has drug cravings, there will be a gap between the actual self and the ought self, which makes the individual extremely prone to negative emotions related to anxiety and depression (Higgins, 1987). In addition, scholars found that an individual drug craving has a significant predictive effect on anxiety and depression, and there is a positive correlation between drug craving and anxiety and depression (Swift and Stout, 1992; Witkiewitz and Bowen, 2010; Xiao et al., 2018; Wolitzky-Taylor and Schiffman, 2019). Therefore, this study hypothesized that drug craving has a mediating effect on anxiety and depression in substance abusers in males (H3).

## Methods

### Design and Participants

A cross-sectional survey was conducted at Yaan Men’s Compulsory Drug Rehabilitation Center, Yaan, Sichuan Province. A total of 600 male substance abusers were recruited from Yaan Men’s Compulsory Drug Rehabilitation Center between May 20 and May 25, 2020. The inclusion criteria were as follows:1) age ≥ 18 years old, 2) Male substance abusers, 3)Yaan Men’s Compulsory Drug Rehabilitation Center managers and doctors assented that the recipient was able to participate in the study, 4) could speak and read Mandarin, and 5) signed an informed consent form for voluntary participation in the study. The exclusion criteria were as follows: diagnosed with severe mental illness or cognitive impairment.

### Methodological Details and Procedures

The male substance abusers in Yaan Men’s Compulsory Drug Rehabilitation Center during the rehabilitation consolidation period were surveyed. The management of the drug rehabilitation center was responsible for issuing questionnaires to male substance abusers. Permission was obtained from the University’s Human Research Ethics Committee. Before administering the questionnaire, participants read the information on the study, and conditions for consent. The information stressed that participation was completely voluntary and anonymous. The survey was conducted within Yaan Men’s Compulsory Drug Rehabilitation Center, it took approximately 20 min to complete the questionnaires. After the questionnaires were completed, they were collected immediately and checked for missing information. Incomplete or incorrect questionnaires would be rejected. A total of 600 questionnaires were distributed in Yaan, fifteen invalid questionnaires were excluded, and a total of 585 valid ones were returned, a response rate of 97.5%. Participants ages ranged between 20 and 58 years (M = 33.21, SD = 6.97). The types of drugs the participants used mainly include Heroin (8%), Cannabis (3%), Cocaine (1%), K powder (5%), Methamphetamines (59%), and Others (24%). Demographic information of substance abusers (see Table 1). Substance abusers’ diagnoses, which were based on the criteria of the Diagnostic and Statistical Manual of Mental Disorders-Fifth Edition (DSM-5; American Psychiatric Association, 2013), were made through treatment team consultation, which included a psychiatrist, a Psychologist, a general physician, and substance use counselors.

**TABLE 1:** Demographic information of substance abusers (*N* = 585).

Items		M ± SD (%)
Sex	Male	585 (100%)
Age(years)		33.21 ± 6.97
Educational level		
	Primary and below	140 (24%)
	Junior middle school	268 (45.8%)
	High school	134 (22.9%)
	Junior college	30 (5.1%)
	College degree and above	13 (2.2%)
Marital status		
	Married	139 (23.8%)
	Unmarried/Divorced/Widowed	446 (76.2%)
Mainly drug used		
	Heroin	47 (8%)
	Cannabis	18 (3%)
	Cocaine	6 (1%)
	K powder	29 (5%)
	Methamphetamine	345 (59%)
	Others	140 (24%)
Average number of drug abuse		
	Less than once a month	54 (9.2%)
	1–3 times a month	141 (24.1%)
	1–2 times a week	126 (21.5%)
	3–4 times a week	108 (18.5%)
	Almost everyday	156 (26.7%)

### Measures

A total of five questionnaires were used in this study.

### General Demographic Questionnaire

A self-developed questionnaire was implemented to obtain information on general demographic variables, such as sex, age, level of education, marital status, mainly drug used, and the average number of drug abuse.

### State-Trait Anxiety Inventory

State and Trait anxiety symptoms were assessed using the State-Trait Anxiety Inventory (STAI) (Spielberger and Gorsuch, 1983), which has been found to be both reliable and valid among china samples (Zheng et al., 1993). The questionnaire has a total of 40 items, consisting of two subscales: State Anxiety Inventory (S-AI) and Trait Anxiety Inventory (T-AI), each containing 20 items. This item was graded on a scale of 1 (not at all) to 4 (very much). Responses for the State Anxiety scale assess intensity of current feelings “at this moment” (e.g., I'm worrying now, and I feel that this kind of worry exceeds the possible misfortune). Responses for the Trait Anxiety scale assess frequency of feelings “in general” (e.g., I worry too much about some things, but in fact they don’t matter). Cronbach’s alpha value for reliability for State-Trait Anxiety Inventory was 0.91 in our sample.

### The UCLA Loneliness Scale

Loneliness was assessed using the UCLA Loneliness Scale [Version 3] (Russell, 1996), Some researchers conducted applicability tests in China (Tan et al., 2016). The UCLA Loneliness Scale comprises 20 items such as “Do you feel close to people?” and “Do you feel left out”. Participants rated each item on a 4-point scale, ranging from 1 (never) to 4 (always). Higher scores represent higher levels of loneliness. In the current study, the internal consistencies of this scale were α = 0.82.

### Drug Craving Scale

The craving belief scale was compiled by Jiang and Lin (2000). The questionnaire has a total of 22 items (such as “Taking drugs can enhance sexual performance, taking drugs is a good way to refresh yourself, and taking drugs can relieve stress”), consisting of three dimensions (e.g., Drug cognition, Irrational belief, and Craving degree). This item was graded on a scale of 1 (strongly disagree) to 5 (strongly agree). This study overall Cronbach’s α coefficient was 0.95.

### The Center for Epidemiologic Studies Depression Scale

The CES-D Scale contains 20 item (Radloff, 1977). The questionnaire included 20 items; one exemplar was “I think the future is full of hope.” This item had to be answered on a scale of 1 (rarely or none of the time) to 4 (most or all the time), with higher scores indicating more depressive symptoms. The internal consistency of the questionnaire was good (α = 0.86).

### Data Analysis

In this study, SPSS 22.0 and AMOS 21.0 were used for data collation and analysis. Statistical methods included reliability, descriptive statistics, and correlation analyses. Descriptive statistics were expressed in percentage, mean, and standard deviation. Spearson correlation analysis was performed to explore the correlations between age, anxiety, loneliness, drug craving, and depression. AMOS 21.0 was used to analyze loneliness and drug craving had significant mediating effects between anxiety and depression. Mediation effect tests were performed according to the two-step procedure recommended by Gerbing and Anderson (1988). If the 95% confidence interval of the mediating effect did not include 0, the mediating effect was significant; otherwise, the mediating effect was not significant. In our research, anxiety was considered the independent variable, depression was considered the dependent variable, loneliness and drug craving were considered the mediating variable to construct the mediating effect model.

## Results

### Correlational Analysis

Means, standard deviations, and correlations among the study variables were presented in Table 2. Specifically, correlation results demonstrated that loneliness, drug craving, and depression were all positively correlated with anxiety; drug craving and depression were all positively correlated with loneliness, age was negatively correlated with loneliness. Besides, drug craving and positively correlated with depression; As such, H1 was confirmed.

**TABLE 2:** Descriptive statistics and correlations (*N* = 585).

Variable	N	M	SD	1	2	3	4	5
1. Age	585	33.21	6.97	−				
2. Anxiety	585	2.38	0.41	−0.06	−			
3. Loneliness	585	2.54	0.42	−0.12**	0.37***	−		
4. Drug craving	585	2.43	0.74	0.07	0.28***	0.16***	−	
5. Depression	585	2.27	0.46	−0.07	0.51***	0.49***	0.39***	−

M = mean; SD = standard deviation.

*p < 0.05; **p < 0.01; ***p < 0.001.

### The Mediating Effect of Loneliness and Drug Craving

Based on existing studies and relationships between variables, we used structural equation modeling (SEM) to analyze the relationships among the variables with anxiety as the exogenous (“independent”) variable and depression as the endogenous (“dependent”) variable after controlling for age. We used a percentile Bootstrap method to repeatedly sample and test for a mediating effect test. If the 95% confidence interval of the mediating effect did not include 0, the mediating effect was significant; otherwise, the mediating effect was not significant. According to the testing procedure of mediating effects (Wen and Ye, 2014), the direct effect of anxiety on depression was to be tested first, followed by the fitness of the model and the significance of each path coefficient after adding in the mediating variable. The direct effect path coefficient of anxiety on depression was significant (*β* = 0.57, *p* < 0.001).

**FIGURE 1:**
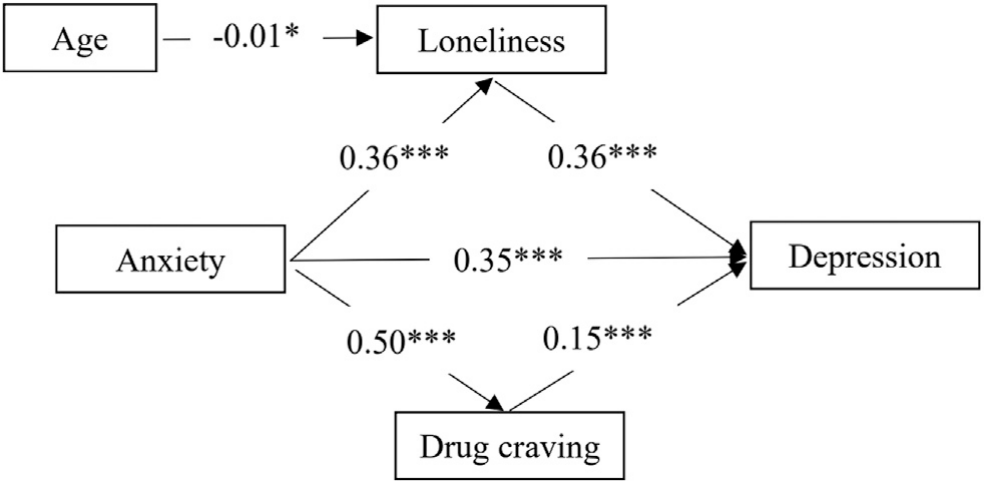
Structural equation model (standardized coefficients). *Notes*. *p < 0.05; **p < 0.01; ***p < 0.001.

We assigned loneliness and drug craving as mediating variables between anxiety and depression. The fitness results were as follows: *χ2/df* = 2.319, RMSEA = 0.048, GFI = 0.997, TLI = 0.971, CFI = 0.994. The results can be seen in Figure 1; Table 3, the path coefficients between anxiety and loneliness (*β* = 0.36, *p* < 0.001), loneliness and depression (*β* = 0.36, *p* < 0.001), age and loneliness (*β* = −0.01, *p* < 0.05), anxiety and drug craving (*β* = 0.50, *p* < 0.001), and drug craving and depression (*β* = 0.15, *p* < 0.001) were significant. However, after adding the mediating variable, the path coefficient between anxiety and depression turned from (*β* = 0.57, *p* < 0.001) decrease to (*β* = 0.35, *p* < 0.001). This study showed that loneliness and drug craving mediated the link between anxiety and depression. Therefore, the results supported H2 and H3.

**TABLE 3:** Intermediate effect bootstrap test.

Path	Standard path effect value	*p* Value
1. Anxiety → loneliness	0.36	< 0.001
2. Loneliness → depression	0.36	< 0.001
3. Age → loneliness	−0.01	< 0.05
4. Anxiety → drug craving	0.50	< 0.001
5. Drug craving → depression	0.15	< 0.001
6. Anxiety → depression	0.35	< 0.001

## Discussion

The present study explored correlations between anxiety, loneliness, drug craving, and depression among Chinese substance abusers in males. However, the psychological factors of substance abusers in female’s depression have not been investigated. Previous studies have found that gender have been found to affect anxiety, loneliness, depression, and craving, male and female have significant differences in these psychological factors (Zilberman et al., 2007; McHugh et al., 2018; Gao et al., 2020; Liu et al., 2020). Thus, future research may also examine how psychological variables (anxiety, loneliness, drug craving, and depression) affect Chinese substance use disorders in females.

This study also investigated the relationship between anxiety and depression in substance abusers in males and its underlying mechanism. The correlation analysis results showed that anxiety and substance abusers in male’s depression were significantly positively correlated, and the research hypothesis H1 was verified. These findings were consistent with previous results on the relationship between anxiety and depression (Panova et al., 2020; Rehman et al., 2021). The mental health of drug users is not only related to the drug itself, but criminal awareness and criminal behavior may also have a significant impact on the mental health of drug users. Many studies have shown that drug users experience problems such as maladjustment and poor mental health (Song, 2002; Ting, 2004; Qu, 2006). Clinically, the comorbidity of anxiety and depression is quite common. Anxiety and depression are both painful and unpleasant emotional experiences, and drug addicts intend to escape them by taking drugs. Previous studies have found that anxiety and depression are the main negative emotions experienced by drug addicts (Lei et al., 2004). After confirming the relationship between anxiety and depression in male drug addicts, it is necessary to explore further whether there is a mediating effect between anxiety and depression in male drug addicts, loneliness, and drug craving. The study results expanded on related research regarding the mechanism of depression in male addicts as well as the intervention in their depression.

Second, this study revealed that loneliness had a significant mediating effect between anxiety and substance abusers in male’s depression and further confirmed Hypothesis 2. Simultaneously, the effect of anxiety on the depression of substance use disorders in males can be realized by directly predicting the depression of substance abusers in males on the one hand and through the mediating effect of loneliness on the other. This was consistent with the results of previous studies; loneliness plays a crucial role in individual development. In different groups, anxiety can affect depression levels by acting on loneliness (Ebesutani et al., 2015). The correlation analysis verified the findings of the predecessors that loneliness has a significant positive predictive effect on anxiety and depression (Ostovar et al., 2016; McHugh Power et al., 2020). Interpersonal theories of depression indicate that positive interpersonal relationships are a protective factor for reducing individual depression (Hammen, 1999) and that autistic patients often show negative interpersonal relationships. Therefore, loneliness has a significant predictive effect on depression and anxiety (Greco and Morris, 2005; Qualter et al., 2010). The results of this study also indicated that loneliness is the main way that anxiety acts on depression, which provides a theoretical basis for targeted and efficient intervention measures, and is significant in alleviating the symptoms of substance abusers in male’s depression and promoting physical and mental health. Therefore, compulsory drug rehabilitation center managers should pay attention to observing the performance of substance abusers in male’s anxiety and loneliness and determine and intervene early to prevent and relieve symptoms of depression effectively.

To clarify how anxiety affects depression in substance use disorders in males, a social cognitive theory (Bandura, 1986) of this research proposes a drug craving mediation model, which helped clarify the mediating mechanism between anxiety and depression in substance abusers in males. This study found that drug craving played a significant mediating role in the relationship between anxiety and substance abusers in males depression. This result supported Hypothesis 3. Drug craving is an important factor that triggers individual negative emotions (anxiety and depression). The relationship between drug cravings, depression, and anxiety has received much research attention (Xiao et al., 2018; Wolitzky-Taylor and Schiffman, 2019), but there are considerably few empirical studies on how drug craving affects depression and anxiety. First, drug addicts with strong drug cravings show a high intensity of motivation toward drugs. The motivational dimensional model of affect (Gable and Harmon-Jones, 2010) suggested that the high intensity of approaching motivation often induces positive emotions, which makes individuals focus on the goal they want to achieve—drugs—and persistently seek them out. When drug users have a strong desire for drugs but cannot obtain them normally, this high intensity of approaching motivation will cause more negative emotions, and anxiety and depression are typical representative emotions (Zou et al., 2011). Second, according to an Affective Processing Model of Negative Reinforcement (Baker et al., 2004), as drugs have a reward-enhancing effect on addicts, drug consumption produces bliss and transcendence. This is the simplest and most effective way for drug addicts to resolve negative emotions; however, it also triggers new and higher levels of anxiety and depression and produces a vicious circle. On this basis, this study confirmed that anxiety could affect substance abusers in male’s depression through the mediating effect of a drug craving. This not only revealed the mechanism of anxiety’s influence on substance abusers in male’s depression but also provides a new perspective for later stage mental health interventions.

Reflecting on the whole process of this research, it was found that the research still had the following limitations: Firstly, with the cross-sectional design, the characteristics of changes in the time course of each variable cannot be obtained. Secondly, the number of survey samples was limited and didn’t have broad representation. Thirdly, only the psychological factors of substance abusers in male’s depression have been investigated, and there was a lack of exploration of its physiological mechanism. Finally, this research only focused on the relationship between anxiety and depression in male drug users and its impact mechanism, and needs to consider other mediating variables, such as self-control, self-efficacy, and self-esteem. These deficiencies should be improved in future research.

## Conclusion

The results indicated that: Loneliness had a significant positive correlation with anxiety and substance abusers in male’s depression; Drug craving had a significant positive correlation with anxiety and substance abusers in male’s depression; Loneliness and drug craving mediated the link between anxiety and substance abusers in male’s depression. In addition, it provided results that may be helpful for the development of new interventional strategies to improve the management of mental health in drug addicts. Overall, this study deepened and expanded the researches about depression, and it provided more clear pathways and strong scientific bases for reducing depression from mood perspective in substance abusers in Males. This study indeed has an important practical significance.

## Data Availability

The data analyzed in this study is subject to the following licenses/restrictions: Please contact the corresponding author for original data. Requests to access these datasets should be directed to cx18996401842@163.com.

## References

[B1] American Psychiatric Association (2013). Diagnostic and Statistical Manual of Mental Disorders (DSM-5®). New York: American Psychiatric Pub

[B2] Asher S. R. Paquette J. A. (2003). Loneliness and Peer Relations in Childhood. Curr. Dir. Psychol. Sci. 12 (3), 75–78. 10.1111/1467-8721.01233

[B3] Bachi K. Sierra S. Volkow N. D. Goldstein R. Z. Alia-Klein N. (2017). Is Biological Aging Accelerated in Drug Addiction? Curr. Opin. Behav. Sci. 13, 34–39. 10.1016/j.cobeha.2016.09.007 27774503 PMC5068223

[B4] Baker T. B. Piper M. E. McCarthy D. E. Majeskie M. R. Fiore M. C. (2004). Addiction Motivation Reformulated: an Affective Processing Model of Negative Reinforcement. Psychol. Rev. 111 (1), 33–51. 10.1037/0033-295x.111.1.33 14756584

[B5] Bandura A. (1986). Social Foundations of Thought and Action: A Social Cognitive Theory. Englewood Cliffs, NJ: Prentice-Hall

[B6] Beesdo K. Bittner A. Pine D. S. Stein M. B. Höfler M. Lieb R. (2007). Incidence of Social Anxiety Disorder and the Consistent Risk for Secondary Depression in the First Three Decades of Life. Arch. Gen. Psychiatry. 64 (8), 903–912. 10.1001/archpsyc.64.8.903 17679635

[B7] Beiter R. Nash R. McCrady M. Rhoades D. Linscomb M. Clarahan M. (2015). The Prevalence and Correlates of Depression, Anxiety, and Stress in a Sample of College Students. J. Affective Disord. 173, 90–96. 10.1016/j.jad.2014.10.054 25462401

[B8] Bekker H. L. Legare F. Stacey D. O’Connor A. Lemyre L. (2003). Is Anxiety a Suitable Measure of Decision Aid Effectiveness: a Systematic Review? Patient Educ. Couns. 50 (3), 255–262. 10.1016/S0738-3991(03)00045-4 12900095

[B9] Bo Y. Xu L. Suyong Y. Shasha A. Liuhua Y. (2007). The Effect of Personality, Social Support and Irrational Belief on the Cravings of Male Drug Abstainers in Reeducation-Through-Labor Institutions. Psychol. Sci. 30 (6), 1413–1417. 10.16719/j.cnki.1671-6981.2007.06.037

[B10] Brundtland G. H. (2001). Mental Health: New Understanding, new hope. Jama. 286 (19), 2391. 10.1001/jama.286.19.2391 11712923

[B11] Cacioppo S. Grippo A. J. London S. Goossens L. Cacioppo J. T. (2015). Loneliness: Clinical Import and Interventions. Perspect. Psychol. Sci. 10 (2), 238–249. 10.1177/1745691615570616 25866548 PMC4391342

[B12] Cami J. Farré M. (2003). Drug Addiction. N. Engl. J. Med. 349 (10), 975–986. 10.1056/NEJMra023160 12954747

[B13] Cardinal R. N. Everitt B. J. (2004). Neural and Psychological Mechanisms Underlying Appetitive Learning: Links to Drug Addiction. Curr. Opin. Neurol. 14 (2), 156–162. 10.1016/j.conb.2004.03.004 15082319

[B14] Chen D. Guan J. Guo Y. M. (2017). The Relationship between Loneliness and Drug Caving of the Addicts: the Mediating Effects of Coping Style. Chin. J Drug Abuse Pre Trea. 1 (4), 22–24. Available at: https://en.cnki.com.cn/Article_en/CJFDTotal-ZYLF201701006.htm.

[B15] Chen X. Qi H. Liu R. Feng Y. Li W. Xiang M. (2021). Depression, Anxiety and Associated Factors Among Chinese Adolescents during the COVID-19 Outbreak: a Comparison of Two Cross-Sectional Studies. Transl Psychiatry. 11 (1), 1–8. 10.1038/s41398-021-01271-4 33654058 PMC7921611

[B16] Cummings C. M. Caporino N. E. Kendall P. C. (2014). Comorbidity of Anxiety and Depression in Children and Adolescents: 20 Years after. Psychol. Bull. 140 (3), 816–845. 10.1037/a0034733 24219155 PMC4006306

[B17] De Los Cobos J. P. Siñol N. Trujols J. Bañuls E. Batlle F. Tejero A. (2011). Drug‐dependent Inpatients Reporting Continuous Absence of Spontaneous Drug Craving for the Main Substance throughout Detoxification Treatment. Drug Alcohol. Rev. 30 (4), 403–410. 10.1111/j.1465-3362.2010.00241.x 21355930

[B18] DeVito A. N. Calamia M. Roye S. Bernstein J. P. Castagna P. (2019). Factor Structure of the Attentional Control Scale in Younger and Older Adults: Relationships with Anxiety and Depression. J. Psychopathol. Behav. Assess. 41 (1), 60–68. 10.1007/s10862-018-9705-3

[B19] Dziedzic B. Sarwa P. Kobos E. Sienkiewicz Z. Idzik A. Wysokiński M. (2021). Loneliness and Depression Among Polish High-School Students. Int. J. Environ. Res. Public Health. 18 (4), 1706. 10.3390/ijerph18041706 33578868 PMC7916597

[B20] Ebesutani C. Fierstein M. Viana A. G. Trent L. Young J. Sprung M. (2015). The Role of Loneliness in the Relationship between Anxiety and Depression in Clinical and School‐based Youth. Psychol. Sch. 52 (3), 223–234. 10.1002/pits.21818

[B21] Erzen E. Çikrikci Ö. (2018). The Effect of Loneliness on Depression: A Meta-Analysis. Int. J. Soc. Psychiatry. 64 (5), 427–435. 10.1177/0020764018776349 29792097

[B22] Gable P. Harmon-Jones E. (2010). The Motivational Dimensional Model of Affect: Implications for Breadth of Attention, Memory, and Cognitive Categorisation. Cogn. Emot. 24 (2), 322–337. 10.1080/02699930903378305

[B23] Gao W. Ping S. Liu X. (2020). Gender Differences in Depression, Anxiety, and Stress Among College Students: a Longitudinal Study from China. J.Affect. Disord. 263, 292–300. 10.1016/j.jad.2019.11.121 31818792

[B24] Gerbing D. W. Anderson J. C. (1988). An Updated Paradigm for Scale Development Incorporating Unidimensionality and its Assessment. J. Mark Res. 25 (2), 186–192. 10.1177/002224378802500207

[B25] Greco L. A. Morris T. L. (2005). Factors Influencing the Link between Social Anxiety and Peer Acceptance: Contributions of Social Skills and Close Friendships during Middle Childhood. Behav. Ther. 36 (2), 197–205. 10.1016/S0005-7894(05)80068-1

[B26] Hamilton J. L. Potter C. M. Olino T. M. Abramson L. Y. Heimberg R. G. Alloy L. B. (2016). The Temporal Sequence of Social Anxiety and Depressive Symptoms Following Interpersonal Stressors during Adolescence. J. Abnorm. Child. Psychol. 44 (3), 495–509. 10.1007/s10802-015-0049-0 26142495 PMC4701637

[B27] Hammen C. (1999). “The Emergence of an Interpersonal Approach to Depression,” in The Interactional Nature of Depression: Advances in Interpersonal Approaches. Editors Joiner T. Coyne J. C. (Washington, DC: American Psychological Association), 21–35.

[B28] Heinrich L. M. Gullone E. (2006). The Clinical Significance of Loneliness: A Literature Review. Clin. Psychol. Rev. 26 (6), 695–718. 10.1016/j.cpr.2006.04.002 16952717

[B29] Herrman H. Kieling C. McGorry P. Horton R. Sargent J. Patel V. (2019). Reducing the Global burden of Depression: a Lancet–World Psychiatric Association Commission. The Lancet. 393 (10189), e42–e43. 10.1016/S0140-6736(18)32408-5 30482607

[B30] Higgins E. T. (1987). Self-discrepancy: a Theory Relating Self and Affect. Psychol. Rev. 94 (3), 319–340. 10.1037/0033-295X.94.3.319 3615707

[B31] Holden C. (2000). Global Survey Examines Impact of Depression. Science. 288 (5463), 39–40. 10.1126/science.288.5463.39 10766633

[B32] Igbokwe C. C. Ejeh V. J. Agbaje O. S. Umoke P. I. C. Iweama C. N. Ozoemena E. L. (2020). Prevalence of Loneliness and Association with Depressive and Anxiety Symptoms Among Retirees in Northcentral Nigeria: a Cross-Sectional Study. BMC Geriatr. 20, 1–10. 10.1186/s12877-020-01561-4 PMC7178938 32326891

[B33] Jiang Z. H. Lin R. Q. (2000). A Study on the Effect of Cognitive Behavior Group Therapy on the Counseling of Drug Abusers. J. Criminol 5, 277–310.

[B34] Jing Y. Xiang Y. Xiang X. Q. (2012). Correlation of Loneliness, Depression and Anxiety Symptoms of the Older In-Patients. Stu Psychol. Behav. 10 (3), 172

[B35] Kara M. Baytemir K. Inceman-Kara F. (2021). Duration of Daily Smartphone Usage as an Antecedent of Nomophobia: Exploring Multiple Mediation of Loneliness and Anxiety. Behav. Inf. Technol. 40 (1), 85–98. 10.1080/0144929X.2019.1673485

[B36] Katz B. A. Matanky K. Aviram G. Yovel I. (2020). Reinforcement Sensitivity, Depression and Anxiety: A Meta-Analysis and Meta-Analytic Structural Equation Model. Clin. Psychol. Rev. 77, 101842. 10.1016/j.cpr.2020.101842 32179341

[B37] Kessler R. C. Stang P. Wittchen H. U. Stein M. Walters E. E. (1999). Lifetime Co-morbidities between Social Phobia and Mood Disorders in the US National Comorbidity Survey. Psychol. Med. 29 (3), 555–567. 10.1017/s0033291799008375 10405077

[B38] Klemanski D. H. Curtiss J. McLaughlin K. A. Nolen-Hoeksema S. (2017). Emotion Regulation and the Transdiagnostic Role of Repetitive Negative Thinking in Adolescents with Social Anxiety and Depression. Cognit. Ther. Res. 41 (2), 206–219. 10.1007/s10608-016-9817-6 PMC5455341 28579659

[B39] Ledford H. (2014). Medical Research: if Depression Were Cancer. Nat. News. 515 (7526), 182–184. 10.1038/515182a 25391943

[B40] Lei W. Li H. L. Jian X. Z. Jia J. T. (2004). An Analysis of Drug Abusers' Psychosocial Life Quality. Psychol. Sci. 27 (2), 284–286. Available at: http://www.cqvip.com/qk/95682a/200402/9485800.html.

[B41] Liu H. Zhang M. Yang Q. Yu B. (2020). Gender Differences in the Influence of Social Isolation and Loneliness on Depressive Symptoms in College Students: a Longitudinal Study. Soc. Psychiatry Psychiatr. Epidemiol. 55 (2), 251–257. 10.1007/s00127-019-01726-6 31115597

[B42] McHugh Power J. Tang J. Kenny R. A. Lawlor B. A. Kee F. (2020). Mediating the Relationship between Loneliness and Cognitive Function: the Role of Depressive and Anxiety Symptoms. Aging Ment. Health. 24 (7), 1071–1078. 10.1080/13607863.2019.1599816 30955348

[B43] McHugh R. K. Votaw V. R. Sugarman D. E. Greenfield S. F. (2018). Sex and Gender Differences in Substance Use Disorders. Clin. Psychol. Rev. 66, 12–23. 10.1016/j.cpr.2017.10.012 29174306 PMC5945349

[B44] Michalska da Rocha B. Rhodes S. Vasilopoulou E. Hutton P. (2018). Loneliness in Psychosis: a Meta-Analytical Review. Schizophrenia Bull. 44 (1), 114–125. 10.1093/schbul/sbx036 PMC5768045 28369646

[B46] Moeller R. W. Seehuus M. (2019). Loneliness as a Mediator for College Students' Social Skills and Experiences of Depression and Anxiety. J. Adolesc. 73, 1–13. 10.1016/j.adolescence.2019.03.006 30933717 PMC6534439

[B47] Nicolaisen M. Thorsen K. (2017). What Are Friends for? Friendships and Loneliness over the Lifespan—From 18 to 79 Years. Int. J. Aging Hum. Dev. 84 (2), 126–158. 10.1177/0091415016655166 27357305

[B48] Ostovar S. Allahyar N. Aminpoor H. Moafian F. Nor M. B. M. Griffiths M. D. (2016). Internet Addiction and its Psychosocial Risks (Depression, Anxiety, Stress and Loneliness) Among Iranian Adolescents and Young Adults: A Structural Equation Model in a Cross-Sectional Study. Int. J. Ment. Health Addict. 14 (3), 257–267. 10.1007/s11469-015-9628-0

[B49] Ottenbreit N. D. Dobson K. S. Quigley L. (2014). An Examination of Avoidance in Major Depression in Comparison to Social Anxiety Disorder. Behav. Res. Ther. 56, 82–90. 10.1016/j.brat.2014.03.005 24727363

[B50] Panova T. Carbonell X. Chamarro A. Puerta-Cortés D. X. (2020). Specific Smartphone Uses and How They Relate to Anxiety and Depression in university Students: a Cross-Cultural Perspective. Behav. Inf. Technol. 39 (9), 944–956. 10.1080/0144929X.2019.1633405

[B51] Qu R. (2006). Drug Addicts' Mental Health Conditions and Their Relations with Relapse Reasons. Chi. J. Clin. Psychol. 14 (1), 55–57. Available at: http://ir.psych.ac.cn/handle/311026/174

[B52] Qualter P. Brown S. L. Munn P. Rotenberg K. J. (2010). Childhood Loneliness as a Predictor of Adolescent Depressive Symptoms: an 8-year Longitudinal Study. Eur. Child. Adolesc. Psychiatry 19 (6), 493–501. 10.1007/s00787-009-0059-y 19777287

[B53] Radloff L. S. (1977). The CES-D Scale: A Self-Report Depression Scale for Research in the General Population. Appl. Psychol. Meas. 1 (3), 385–401. 10.1177/014662167700100306

[B54] Rehman U. Shahnawaz M. G. Khan N. H. Kharshiing K. D. Khursheed M. Gupta K. (2021). Depression, Anxiety and Stress Among Indians in Times of Covid-19 Lockdown. Community Ment. Health J. 57, 42–48. 10.1007/s10597-020-00664-x 32577997 PMC7309680

[B55] Richardson T. Elliott P. Roberts R. (2017). Relationship between Loneliness and Mental Health in Students. J. Public Ment. Health 16 (2), 48–54. 10.1108/JPMH-03-2016-0013

[B56] Robinson T. E. Berridge K. C. (1993). The Neural Basis of Drug Craving: an Incentive-Sensitization Theory of Addiction. Brain Res. Rev. 18 (3), 247–291. 10.1016/0165-0173(93)90013-P 8401595

[B57] Russell D. W. (1996). UCLA Loneliness Scale (Version 3): Reliability, Validity, and Factor Structure. J. Pers. Assess. 66 (1), 20–40. 10.1207/s15327752jpa6601_2 8576833

[B58] Santini Z. I. Jose P. E. Cornwell E. Y. Koyanagi A. Nielsen L. Hinrichsen C. (2020). Social Disconnectedness, Perceived Isolation, and Symptoms of Depression and Anxiety Among Older Americans (NSHAP): a Longitudinal Mediation Analysis. The Lancet Public Health. 5 (1), e62–e70. 10.1016/S2468-2667(19)30230-0 31910981

[B59] Self D. W. (1998). Neural Substrates of Drug Craving and Relapse in Drug Addiction. Ann.Med. 30 (4), 379–389. 10.3109/07853899809029938 9783837

[B60] Smith K. (2014). Mental Health: a World of Depression. Nat. News. 515 (7526), 180. Available at: https://www.nature.com/news/mental-health-a-world-of-depression-1.16318 10.1038/515180a 25391942

[B61] Song Z. Y. (2002). A Study on Personality Characteristics and Personality Types of Drug Users. Chi. J. Clin. Psychol. 10 (3), 224–226.

[B62] Spielberger C. D. Gorsuch R. L. (1983). State-trait Anxiety Inventory for Adults: Manual and Sample: Manual, Instrument and Scoring Guide. Consulting Psychologists Press

[B63] Swift R. M. Stout R. L. (1992). The Relationship between Craving, Anxiety, and Other Symptoms in Opioid Withdrawal. J. Subst. Abuse. 4 (1), 19–26. 10.1016/0899-3289(92)90024-R 1320971

[B64] Tan J. Ai Y. Wen X. Wu Y. Wang W. (2016). Relationship between Shyness and Loneliness Among Chinese Adolescents: Social Support as Mediator. Soc. Behav. Pers. 44 (2), 201–208. 10.2224/sbp.2016.44.2.201

[B65] Ting H. Y. L. (2004). The Investigation and Analysis of Interpersonal Relationship and Self-Esteem on 242 Cases of Female Drug Abstainers. Psychol. Expl. 24 (2), 63–66. 10.3969/j.issn.1003-5184.2004.02.014 . Available at: https://en.cnki.com.cn/Article_en/CJFDTotal-XLXT200402014.htm

[B66] Vos T. Abajobir A. A. Abate K. H. Abbafati C. Abbas K. M. Abd-Allah F. (2017). Global, Regional, and National Incidence, Prevalence, and Years Lived with Disability for 328 Diseases and Injuries for 195 Countries, 1990–2016: a Systematic Analysis for the Global Burden of Disease Study 2016. The Lancet. 390 (10100), 1211–1259. 10.1016/S0140-6736(17)32154-2 PMC5605509 28919117

[B67] Wang Y. H. Li J. Q. Shi J. F. Que J. Y. Liu J. J. Lappin J. M. (2020). Depression and Anxiety in Relation to Cancer Incidence and Mortality: a Systematic Review and Meta-Analysis of Cohort Studies. Mol. Psychiatry. 25 (7), 1487–1499. 10.1038/s41380-019-0595-x 31745237

[B68] Wang Y. Tian L. Guo L. Huebner E. S. (2020). Family Dysfunction and Adolescents' Anxiety and Depression: A Multiple Mediation Model. J. Appl. Dev. Psychol. 66, 101090. 10.1016/j.appdev.2019.101090

[B69] Weiss R. S. (1987). Reflections on the Present State of Loneliness Research. J. Soc. Behav. Pers. 2 (2), 1–16. Available at: https://psycnet.apa.org/record/1988-26577-001.

[B70] Wen Z. Ye B. (2014). Analyses of Mediating Effects: The Development of Methods and Models. Adv. Psychol. Sci. 22 (5), 731–745. 10.3724/SP.J.1042.2014.00731

[B71] WHO (2018). Depression.Retrieved from http://www.who.int/news-room/fact-sheets/detail/depression

[B72] Witkiewitz K. Bowen S. (2010). Depression, Craving, and Substance Use Following a Randomized Trial of Mindfulness-Based Relapse Prevention. J. Consult. Clin. Psychol. 78 (3), 362–374. 10.1037/a0019172 20515211 PMC3280693

[B73] Wolitzky-Taylor K. Schiffman J. (2019). Predictive Associations Among the Repeated Measurements of Anxiety, Depression, and Craving in a Dual Diagnosis Program. J. Dual. Diagn. 15 (3), 140–146. 10.1080/15504263.2019.1589660 30982462

[B74] Xiao Q. Z. Xiang X. Sheng H. D. (2018). Drug Craving and Relapsing Tendency: the Multiple Mediating and Regulatory Effects. Chi. J. Clin. Psychol. 26 (5), 947–951. 10.16128/j.cnki.1005-3611.2018.05.024

[B75] Yang X. Zhou Z. Liu Q. Fan C. (2019). Mobile Phone Addiction and Adolescents’ Anxiety and Depression: the Moderating Role of Mindfulness. J. Child. Fam. Stu. 28 (3), 822–830. 10.1007/s10826-018-01323-2

[B76] Zheng X. H. Shu L. Zhao J. F. (1993). Test Report of State-Trait Anxiety Questionnaire in Changchun. Chi. Men. Health J. 7 (2), 60–62.

[B77] Zilberman M. L. Tavares H. Hodgins D. C. El-Guebaly N. (2007). The Impact of Gender, Depression, and Personality on Cravings. J. Addict. Dis. 26 (1), 79–84. 10.1300/J069v26n01_10 17439871

[B78] Zou J. L. Zhang X. C. Zhang H. Yu L. Zhou R. L. (2011). Beyond Dichotomy of Valence and Arousal: Review of the Motivational Dimensional Model of Affect. Adv. Psychol. Sci. 19 (9), 339–346. Available at: http://journal.psych.ac.cn/xlkxjz/EN/

